# Patterns and influencing factor of synonymous codon usage in porcine circovirus

**DOI:** 10.1186/1743-422X-9-68

**Published:** 2012-03-15

**Authors:** Xin-sheng LIU, Yong-guang Zhang, Yu-zhen Fang, Yong-lu Wang

**Affiliations:** 1 State Key Laboratory of Veterinary Etiological Biology, National Foot and Mouth Disease Reference Laboratory, Lanzhou Veterinary Research Institute, Chinese Academy of Agricultural Sciences, Lanzhou 730046, People's Republic of China

## Abstract

**Background:**

Analysis of codon usage can reveal much about the molecular evolution of the viruses. Nevertheless, little information about synonymous codon usage pattern of porcine circovirus (PCV) genome in the process of its evolution is available. In this study, to give a new understanding on the evolutionary characteristics of PCV and the effects of natural selection from its host on the codon usage pattern of the virus, Patterns and the key determinants of codon usage in PCV were examined.

**Methods:**

We carried out comprehensive analysis on codon usage pattern in the PCV genome, by calculating relative synonymous codon usage (RSCU), effective number of codons (ENC), dinucleotides and nucleic acid content of the PCV genome.

**Results:**

PCV genomes have relatively much lower content of GC and codon preference, this result shows that nucleotide constraints have a major impact on its synonymous codon usage. The results of the correspondence analysis indicate codon usage patterns of PCV of various genotypes, various subgenotypes changed greatly, and significant differences in codon usage patterns of Each virus of Circoviridae.There is much comparability between PCV and its host in their synonymous codon usage, suggesting that the natural selection pressure from the host factor also affect the codon usage patterns of PCV. In particular, PCV genotype II is in synonymous codon usage more similar to pig than to PCV genotype I, which may be one of the most important molecular mechanisms of PCV genotype II to cause disease. The calculations results of the relative abundance of dinucleotides indicate that the composition of dinucleotides also plays a key role in the variation found in synonymous codon usage in PCV. Furthermore, geographic factors, the general average hydrophobicity and the aromaticity may be related to the formation of codon usage patterns of PCV.

**Conclusion:**

The results of these studies suggest that synonymous codon usage pattern of PCV genome are the result of interaction between mutation pressure and natural selection from its host. The information from this study may not only have theoretical value in understanding the characteristics of synonymous codon usage in PCV genomes, but also have significant value for the molecular evolution of PCV.

## Background

Genetic information is transmitted from mRNA to protein in a mode of triplet codon. Each amino acid matches with at least one codon, at most six codons. The codons encoding the same amino acid is called synonymous codon. During biosynthesis of protein, usage probability of those synonymous codons is different. Some species or some genes are usually prone to use one or several particular synonymous codons. These codons are called preferable codons, which is called as codon bias. Usage bias of codons from various species has been studied, and it is found that during protein biosynthesis synonymous codons encoding amino acid is not used randomly [1-3]. Many studies have indicated that obvious bias exists between different genes from different species or the same species [4-6]. Usage bias of codons is influenced mainly by mutation bias, translation selection, secondary protein structure, replication and selective transcription, hydrophobia and hydrophilia of protein, and external environment [7-13].

PCV belongs to genus of porcine circovirus, family of porcine circovirus. It has two genotype, PCV genotype I and PCV genotype II, and it is the smallest virus which has been discovered so far [14]. Among the different genotype, PCV genotype II infection and its related diseases have become one big problem across the globe for pig feeding, which threatens greatly to normal development of the industry of pig feeding. The PCV genome is a single-stranded negative circular DNA, and very small; full length of the PCV genotype I is only 1,759 bp, and PCV genotype II, 1,767 bp or 1,768 bp. The genome contains 11 open reading frames (ORF), among which, ORF1 encodes replication-associated proteins (Rep and Rep'); ORF2, structural proteins (viral capsid proteins, Cap); ORF3, toxicity-associated proteins, which can cause apoptosis [15,16]. By analyzing the whole sequence of PCV genome, it is found that ORF2 has smaller selective pressure than ORF1, and more mutation. Nucleotide sequences among various strains in the same genotype are very conservative, their homology is over 90%, while similarity between nucleotide sequences from various strains respectively from the two genotypes is less than 80% [17,18]. However, so far, studies have not related to usage of PCV codons. Explanation of codon usage pattern of PCV has significance on PCV evolution, gene prediction, gene classification, design of high expressed genes and viral vectors, and understanding of interaction between PCV and its host cells. Therefore, in this study, we first performed comprehensive analysis on codon usage pattern of PCV genome and the related factors affecting on codon usage. This study will play a major role in explanation of evolution process of PCV genome and further studies.

## Results

### The characteristics of synonymous codon usage in PCV

**Figure 1:**
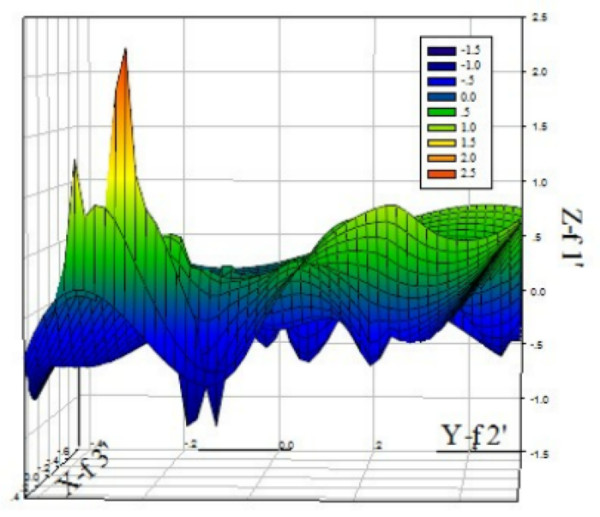
**Three-dimension mesh plot of correspondence analysis value of 59 synonymous codons in PCV**. It can be seen from the plot, change range on the Z axis(*f*'1)s from 15 to 2.5, which indicates PCV codon usage is not balanced.

In order to investigate usage pattern of the PCV codons, we calculated various RSCU values of various codons from 28 different strains from different genotype. It can be seen from the three-dimension mesh plot of the analysis results of correspondence between 59 synonymous codons in the PCV genome (See Figure 1), range of Z axis (*f*'1) is between 15 to 2.5, which indicates that synonymous codons usage in the PCV genome is not balanced, that is to say, among all the 59 synonymous codons, a part of codon is rarely used, while others have higher usage frequency. Additionally, it can be seen from Table 1 that among 18 preferable codons, 12 ones have the end base of G or U, only 6 have the end base of A or C, and so those codons with the end base of G or U are prone to use in PCV genome. Nevertheless, compared with other vertebrate DNA viruses, PCV genome has lower GC%, from 48.35% to 49.12%, with an average content of 48.61% and SD value of 0.19 (Table 2). And hence, the phenomenon, that in PCV genome GC content is lower while the condons with the end base of G is used in a way of bias, suggests that content of G or C as the end base of codons has effect on usage pattern of synonymous codons. Apart from this, we can also see from Table 2, ENC values between PCV genomes has less fluctuation, with a range from 55.32 to 58.67, and an average value of 56.80 and SD value of 0.85, which indicates that codon bias of the PCV genome is stable.

**Table 1:** The relative synonymous codon usage frequency (RSCU) of PCV and swine

*AA^a^*	*Codon*	*SUS^b^*	*PCV*	*AA^a^*	*Codon*	*SUS^b^*	PCV
Leu	UUA	0.65	0.957	Tyr	UAU	**1.12**	0.964
	UUG	0.85	1.372		UAC	0.82	**1.036**
	CUU	1.20	0.912	His	CAU	0.97	**1.078**
	CUC	1.12	0.761		CAC	**1.03**	0.921
	CUA	0.56	0.465	Gln	CAA	0.85	0.733
	**CUG**	**1.62**	**1.528**		**CAG**	**1.15**	**1.266**
Ile	AUU	1.06	**1.441**	Asn	**AAU**	**1.02**	**1.087**
	AUC	**1.11**	0.786		AAC	0.98	0.912
	AUA	0.83	0.769	Lys	AAA	**1.21**	0.907
Val	GUU	1.11	0.875		AAG	0.79	**1.092**
	GUC	0.96	0.606	Asp	GAU	0.95	0.905
	GUA	0.64	1.220		**GAC**	**1.05**	**1.094**
	**GUG**	**1.29**	**1.297**	Glu	**GAA**	**1.09**	**1.348**
Gly	GGU	0.81	1.016		GAG	0.91	0.651
	GGC	1.08	0.632	Cys	**UGU**	**1.06**	**1.275**
	GGA	**1.18**	1.118		UGC	0.94	0.724
	GGG	0.94	**1.232**	Arg	CGU	0.55	0.572
Pro	CCU	**1.26**	1.061		CGC	0.65	0.376
	CCC	1.08	1.099		CGA	0.54	0.704
	CCA	1.23	**1.274**		CGG	0.74	1.102
	CCG	0.43	0.566		**AGA**	**1.86**	**1.842**
Thr	ACU	1.19	1.127		AGG	1.67	1.400
	ACC	1.23	**1.401**	Ser	AGU	0.93	**1.322**
	ACA	**1.24**	0.682		AGC	1.22	1.310
	ACG	0.34	0.785		UCU	**1.34**	1.064
Ala	GCU	**1.36**	0.699		UCC	1.20	1.067
	GCC	1.22	1.027		UCA	1.00	0.812
	GCA	1.05	1.118		UCG	0.31	0.419
	GCG	0.37	**1.153**	Phe	**UUU**	**1.11**	**1.402**
					UUC	0.89	0.597

a AA is the abbreviation of Amino Acid.

b SUS is swine.

c The preferentially used codons for each amino acid are displayed in bold.

**Table 2:** Nucleotide content of 28 PCV genomes

No	A%	U%	G%	C%	A_3_%	U_3_%	G_3_%	C_3_%	GC%	GC_3_%	ENC	Gravy	Aromo
1	24.56	27.00	28.99	19.44	28.32	27.50	23.40	20.01	48.44	43.41	55.76	-0.233824	0.134191
2	24.45	26.95	28.88	19.73	27.47	28.00	25.10	20.00	48.61	45.10	56.45	-0.107513	0.109123
3	24.50	26.83	28.94	19.74	28.49	27.10	23.20	20.00	48.66	43.20	56.13	-0.239154	0.134191
4	24.39	26.78	28.99	19.84	19.79	31.20	28.50	20.00	48.83	48.50	56.52	-0.18045	0.107266
5	24.45	26.89	28.94	19.73	28.49	27.30	23.20	20.02	48.66	43.22	55.74	-0.246507	0.130515
6	24.45	27.00	28.99	19.56	28.15	27.50	23.40	20.00	48.55	43.40	56.21	-0.239338	0.134191
7	24.56	26.89	28.99	19.56	28.32	27.50	23.40	20.01	48.49	43.41	56.3	-0.230092	0.13211
8	24.45	27.06	28.94	19.56	28.15	27.50	23.40	20.01	48.49	43.41	55.97	-0.221771	0.132841
9	24.50	26.89	28.99	19.61	19.96	31.60	28.20	20.00	48.61	48.20	56.16	-0.184256	0.108997
10	25.57	25.90	28.11	20.24	26.48	24.60	27.70	20.00	48.53	47.70	57.36	-0.188909	0.100179
11	25.62	25.90	28.00	20.84	24.44	25.00	28.90	20.00	48.47	48.90	56.87	0.097557	0.08377
12	25.30	25.98	28.35	20.32	25.00	25.00	28.90	20.00	48.67	48.90	57.64	0.102817	0.09507
13	25.85	25.57	28.45	20.14	24.95	24.80	28.90	20.05	48.59	48.95	57.18	0.055789	0.091228
14	25.51	26.07	28.00	20.42	26.48	24.40	27.80	20.00	48.42	47.80	57.33	-0.173298	0.098566
15	25.57	25.96	28.05	20.42	25.97	26.10	29.50	20.00	48.47	49.50	57.37	-0.1475	0.089286
16	25.57	26.07	28.00	20.36	26.65	24.40	27.80	20.00	48.36	47.80	57.8	-0.175269	0.102151
17	25.51	25.96	28.11	20.24	28.18	22.20	31.40	20.00	48.53	51.40	55.32	-0.247464	0.119565
18	25.57	26.19	27.94	20.31	32.42	25.50	28.40	20.00	48.25	48.40	56.1	0.119264	0.085814
19	25.53	26.12	28.62	20.36	28.35	22.10	31.20	20.02	48.42	51.22	55.53	-0.219601	0.117967
20	25.92	25.30	27.50	21.28	25.29	25.0	27.80	20.00	48.78	47.80	58.67	0.044658	0.084063
21	25.35	25.98	28.30	20.73	24.95	25.10	22.80	20.00	48.67	42.80	57.64	0.096485	0.093146
22	25.41	25.69	28.47	20.43	24.44	25.10	29.00	20.00	48.90	49.00	57.56	0.052539	0.087566
23	25.35	26.03	28.24	20.37	24.95	25.10	28.90	20.02	48.61	48.92	57.7	0.1	0.093146
24	25.41	25.98	28.18	20.43	27.67	22.90	30.60	20.00	48.61	50.60	56.08	-0.276311	0.113924
25	24.34	26.54	28.81	20.32	26.48	28.20	23.60	20.01	49.12	43.61	57.44	-0.089698	0.106572
26	24.34	26.54	28.81	20.32	26.48	28.20	23.60	20.00	49.12	43.60	57.44	-0.089698	0.106572
27	25.30	26.15	28.24	20.32	24.95	25.10	28.90	20.00	48.56	48.90	57.82	0.104921	0.096661
28	25.30	26.09	28.24	20.37	27.67	22.90	30.60	20.00	48.61	50.60	56.42	-0.257895	0.112523

### Nucleotide content of all PCV genomes

Natural selection and mutation pressure has been considered to be two key factors which have effect on codon usage patterns of organisms [19]. In order to explore whether determinative factors for codon usage mutation in PCV is mutation pressure or natural selection, we compared correlation between A_3_%, U_3_%, G_3_%, C_3_%, GC_3_% and U_3_%, G_3_%, C_3_%, GC_3_% with correlation analysis (Table 3). The analysis results show, except GC%, GC3% has marked correlation with A%, U%, G% and C%. This indicates that GC_3_% can reflect interaction between natural selection and mutation pressure to some extent.

**Table 3:** The correlation analysis between the A, U, C, G contents and A_3_, U_3_, C_3_, G_3 _contents in all ORF of PCVa

	*A_3_%*	*U_3_%*	*G_3_%*	*C_3_%*	GC_3_%
A%	-0.133^NS^	0.747***	0.499**	-0.114^NS^	0.509**
U%	0.459 *	0.682***	-0.572*	0.222^NS^	-0.578***
G%	0.130^NS^	0.696***	-0.467*	0.362^NS^	-0.460*
C%	-0.416*	-0.566**	0.475*	-0.327^NS^	-0.463*
GC%	0.395*	0.353^NS^	0.151^NS^	-0.103^NS^	-0.152^NS^

a Value in this table is the R value of correlation analysis.

***P-value < 0.001.

**P-value < 0.01.

*0.01 P-value 0.05

NS in superscript represent non-significant

In addition, the correlation between f'1,f'2 value and A%, U%, G%,C%, GC%, A_3_%, U_3_%, G_3_%, C_3_%, GC_3_% was analyzed (Table 4). The results showed that significant correlations exist between synonymous codon usage pattern and nucleotide composition in PCV. This result further verifies the conclusion that during the shaping of synonymous codon usage pattern of PCV, Composition constraints play and very important role.

**Table 4:** The correlation analysis between the first two axes in CA and the nucleotide contents of PCV^a b^

	*A%*	*U%*	*G%*	*C%*	*GC%*	*A_3_%*	*U_3_%*	*G_3_%*	*C_3_%*	GC_3_%
**f'_1_'**	0.406*	-0.592***	-0.458*	0.557**	0.146***^NS^***	-0.735***	-0.088***^NS^***	0.398*	-0.202***^NS^***	0.389*
*f'*_2_*'*	-0.094^NS^	-0.135^NS^	0.005^NS^	0.262^NS^	0.351^NS^	-0.222^NS^	-0.074^NS^	0.603**	-0.177^NS^	0.591**

a Value in this table is the R value of correlation analysis.

b *f*'_1 _and *f'*_2 _, respectively, represent the values of the first and the second axis of each gene in CA.

NS in superscript represent non-significant

***P-value < 0.001.

**P-value < 0.01.

*0.01 < P-value < 0.05

### Genetic relationship based on synonymous codon usage

**Figure 2:**
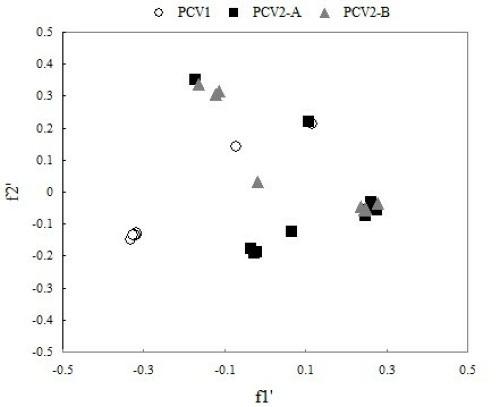
**Correspondence analysis plot of relative synonymous codon usage in PCV**. The first dimension variable *f'*_1 _and the second variable *f'*_2 _can reflect 39.87% and 23.83% of total mutation respectively.

In order to compare synonymous codon usage patterns between different PCV genomes from different genotype, we carried out analysis on codon usage of different PCV genotype with correspondence analysis (CA). In correspondence analysis, the first dimension variable *f'*1 and the second dimension variable *f'*2 can reflect 39.87 and 23.83 percent of total mutation respectively. We can see from Figure 2, except for two strains of PCV genotype I in deviation from the cluster, other all the strains of PCV genotype II lie in the same cluster and overlap partly with each other. Obviously, PCV genotype I and PCV genotype II lay in two independent areas, which demonstrate that codon usage between the different PCV genotype is of great significance. Meanwhile, PCV genotype II-A and PCV genotype II-B almost lie in the same area, but they have tiny difference (Figure 2), which suggests that different sub-genotype from the same PCV genotype have difference in the aspect of codon usage.

### Relationships between codon usage pattern of PCV and that of the host

**Figure 3:**
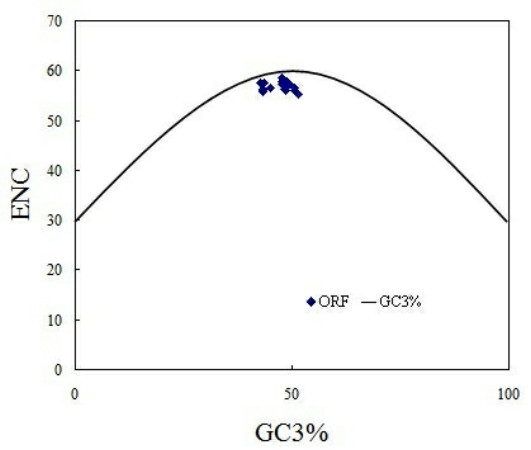
**The relationship between the effective number of codons (ENC) and the GC content of the third codon position (GC_3_%)**.

**Figure 4:**
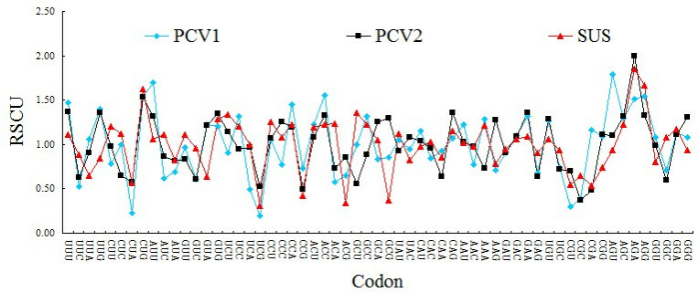
**Compare the codon the codon usage pattern among PCV genotype I, PCV genotype II and swine**.

From ENC values and corresponding relation distribution diagram of GC_3_% (Figure 3) we can see, most points are near or under the theoretical curve, which suggests that apart from mutation pressure which influences on codon usage pattern in PCV, the usage pattern is also influenced by other factors. As parasitic organisms, virus's codon usage pattern would be subject to its host to some extent [6]. In this study, patterns of codon usage are compared in PCV and its natural host, and then found that there are high similarities between them. In detail, the high frequently used codons in the swine were also the non-preferred codons of PCV, such as CUG, GUG, CAG, AAU, GAC, GAA, UGU, AGA and UUU. Further more, all preferentially used codons of the genome of PCV and swine were all G-ended or U-ended codons (Table 1). These results suggest that the selection pressure from the host affects codon usage pattern of PCV. It is worthwhile to note that PCV genotype II have high similarities with swine than PCV genotype I (Figure 4). In details, the values of RSCU in PCV genotype II and swine codon such as, AGA for Arg, GUG for Val, AGC for Ser, ACC for Thr were clearly different from that of PCV genotype I. It may be important one of Molecular mechanisms of infection and pathogenesis of PCV genotype II.

### Relationship between dinucleotide biases and codon usage in PCV

Researches in recent years indicated that dinucleotide biases can affect codon bias [20]. To study the possible effect of dinucleotide composition on codon usage of the PCV genome, the relative abundances of the 16 dinucleotides in genome of the 28 PCV strains were calculated (Table 5). The result show that the occurrences of dinucleotides are not randomly distributed and no dinucleotides were present at the expected frequencies. The relative abundance of CpG showed a strong deviation from the "normal range" (mean ± SD = 0.622 ± 0.029) and was markedly under represented. Correspondingly, the synonymous codon containing the CpG were inhibited because of CpG was present at the expected frequencies. In detail, low RSCU values were present in 7 of all 8 codon containing CpG and they are all not preferentially used codons (such as CCG, UCG, ACG, CGC, CGG, CGA, CGU) (Table 1). These observations indicate that the composition of dinucleotides also plays a key role in the variation found in synonymous codon usage among PCV.

**Table 5:** Relative abundance of dinucleotides in PCV

		Relative abundance of the 16 dinucleotides						
	**AA**	**AU**	**AG**	**AC**	**UU**	**UG**	**UC**	**UA**
Mean ± SD^a^	0.924 ± 0.053	0.870 ± 0.032	1.045 ± 0.029	0.905 ± 0.085	0.881 ± 0.043	1.059 ± 0.037	0.852 ± 0.063	0.826 ± 0.028
	GG	GC	GA	GU	CC	CA	CU	CG
Mean ± SD^a^	0.887 ± 0.042	0.927 ± 0.044	0.890 ± 0.027	0.887 ± 0.033	1.105 ± 0.056	1.046 ± 0.097	1.051 ± 0.029	0.622 ± 0.029

a Mean values of 28 PCV genomes relative dinucleotide ratios ± standard deviation.

### Synonymous codon usage in different viruses of circoviridae is virus specific

**Figure 5:**
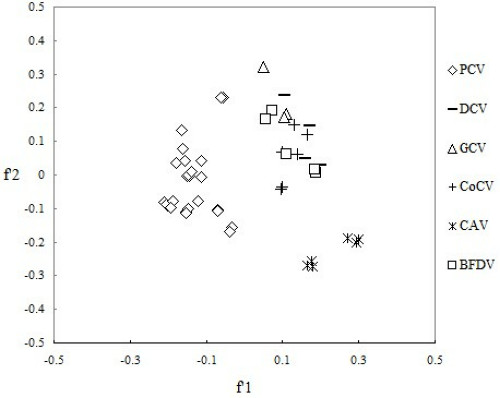
**Correspondence analysis plot of relative synonymous codon usage in Circoviridae **. Each virus strain was divided by species.

The correspondence analysis has been performed in order to compare the synonymous codon usage pattern between the viruses of Circoviridae. From which we could detect one major trend in the f'1 which accounted for 20.27% of the total variation, and another major trend in the f'1 for 15.46%of the total variation. A plot of the f'1 and the f'1 of each virus of Circoviridae was shown in Figure 5. We can see from the plot that there were considerable differences for codon usage patterns among PCV, DCV, GCV, CoCV, CAV and BFDV. Concretely, PCV belong to the different genotype tends to come together. Moreover, DCV, GCV, CoCV and BFDV tend to come together and CAV was alone in separate area. It's clear that although different virus tends to come together, differences of synonymous codon usage pattern still exist between each virus. Therefore, the synonymous codon usage pattern of each virus of Circoviridae varies by the species of virus.

### Effect of other factors on codon usage

**Figure 6:**
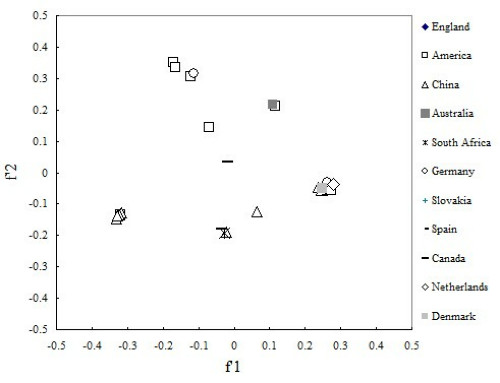
**A plot of value of the first and second axis of each PCV strain for correspondence analysis**. Each PCV strain was divided by geographical area.

To investigate whether there is a correlation between the codon usage of PCV and geographic factor, 28 virus genes of PCV were divided into eleven groups according to obtained area, and correspondence analysis was also used. As can be seen from the plot, coordinate of virus isolates from different country is separated, and these relatively isolated spots tend to cluster into several groups according to the genotype (Figure 6). All above imply that these strains of PCV isolated from different places have different trend in codon usage variation. In addition, we performed another correlation analysis on f'1 in principal component analysis between GRAVY and the aromaticity score of each protein. From the result, we found that f'1 was high positive correlation with GRAVY (Spearman, r = 0.875, *p *< 0.001), and high negative correlation with aromaticity (Spearman, r = 0 905 *p *< 0.001).

## Discussion

During protein biosynthesis synonymous codon encoding amino acids are not used randomly, and some species or some gene always prefers to use of one or several particular synonymous codons, which is called as codon usage bias. Precious studies reveal that different genes from different species or the same one have obvious codon usage bias [21,22]. Codon usage bias is influenced mainly by mutation bias [23,24], translation selection [25,26], secondary protein structure [20,27], replication and transcription selection [28], secondary mRNA structure [29], gene length [30], tRNA abundance [31], gene function and external environment [32]. However, most of these studies focus on some higher organism and many microorganisms with large genome and more genes, and there are few studies on virus with small genome and few genes or comparison between virus and host. Relatively, there are more reports on codon usage in genomes from viruses with great harm to mankind, such as SARS, human immunodeficiency virus, influenza virus A and hepatitis virus. PCV is a primary pathogen of postweaning multisystemic wasting syndrome (PMWS), which has threatened the development of pig feeding industry seriously because in recent year's occurrence of this disease has increased so as to bring about great economic loss in the world industry of pig feeding. Further studies on codon usage pattern in PCV have great significance on mutation pattern and molecular evolution of PCV. However, reports on codon usage pattern in PCV are rare, and this study is the first report.

By comparison with reported DNA viruses such as Duck plague virus, Duck enteritis virus, Iridovirus, Herpesvirus [33-36], synonymous codon usage bias in the PCV genome is low at large (average ENC values is 56.80, and minimum is 55.). This suggests that low codon bias may result from increase in itself replication efficiency in PCV in order to adapt to replication system of its hosts.

In this study, relation between main indices (f'1 and f'2)for the correspondence analysis on PCV usage cofon usage and its nucleotide composition (See Table 2) indicates, mutation pressure has a significant role in PCV codon usage. Other factors which can influence on PCV codon usage are also analysed and the initial results show that mutation pressure is the main factor to influence on PCV codon usage variation.

There were reports that natural selection can influence on synonymous codon usage pattern in viruses and the same conclusions are also obtained from this study. Three evidences support this conclusion. The first evidence is that PCV genome is GC3% -poor (average value = 47.08, SD = 2.88), but most of preferentially used codons are G/T-ended codons. Meanwhile, average of A_3_% is higher than that of T_3_%, but among the codons which PCV prefers to using, there are only three preferable codons with the end base of A_3_% while six those with the end base of T_3_%. The second evidence is that the high similarities exist between PCV and its natural host. The third evidence is that CpG and the synonymous codon including it were inhibited. The three above evidences both state that natural selection is involved in formation of PCV synonymous codon usage pattern.

At present, according to pathogenicity, antigenicity and nucleotide sequence difference, PCV is divided into two genotypes, PCV genotype I and PCV genotype II, of which PCV genotype II includes various subtypes. From significance of PCV codon usage between different genotypes in Figure 1, we can see that PCV codon bias may have association with genotypes. In addition to this, the results in this study also reveal that geological factor may almost have relation with codon usage in PCV. In some reports, gene length has certain correlation with codon usage [30]. Similarly, in some viruses, gene length has no effect on codon usage [22]. With correlation analysis we surveyed codon usage bias and gene length in PCV, and it is found that in these viral genes, codon usage bias has no notable correlation with gene length (Spearman, r = 0.075, *p *> 0.1). The results indicate that PCV gene length has no effect on synonymous codon usage. Other factors, including GRAVY and aromaticity may also significantly influence codon usage of PCV

## Conclusions

Taken together, the codon usage patterns of PCV possibly result from interactions between natural selection and mutation pressure. These results not only provide an insight into the variation of codon usage pattern among the genomes of PCV, but also may help in understanding the processes governing the evolution of PCV.

## Materials and methods

### Sequence data

The information of 28 PCV genomes, including the genotype, length value, the isolated area and GenBank accession numbers of these strains was listed in the Table 6. In order to compare the differences between PCV and its host, twenty swine gene were gained and detailed information of these genes is listed in Table 7. In addition, to compare the codon usage patterns among different viruses, twenty-five viral genomes of Circoviridae were taken into account (Table 8). All of the sequences were downloaded from NCBI (http://www.ncbi.nlm.nih.gov/Genbank/). Each general nucleotide composition (T%,A%,C% and G%) and each nucleotide composition in the third site of codon (T_3_%,A_3_%,C_3_% and G_3_%) in PCV coding sequence were calculated by biosoftware DNAStar7.0 for windows.

**Table 6:** PCV genome sequences included in this study

*No*	*Genotype*	*Length (bp)*	*Isolation*	Accession no
1	PCVI	1759	UK	U49186
2	PCVI	1759	USA	AY099501
3	PCVI	1759	USA	AY184287
4	PCVI	1759	USA	HM143844
5	PCVI	1759	China	GU722334
6	PCVI	1759	China	DQ650650
7	PCVI	1759	China	DQ472015
8	PCVI	1759	China	AY660574
9	PCVI	1759	Australia	AY754015
10	PCVII-a	1768	South Africa	AY325495
11	PCVII-a	1768	Germany	AF201305
12	PCVII-a	1768	Slovakia	HM009338
13	PCVII-a	1768	Spain	AF201308
14	PCVII-a	1768	China	AF381175
15	PCVII-a	1768	China	AY288135
16	PCVII-a	1768	Canada	AF027217
17	PCVII-a	1768	USA	AY099499
18	PCVII-a	1768	USA	AF264042
19	PCVII-a	1768	USA	AY099496
20	PCVII-b	1767	China	EF619037
21	PCVII-b	1767	China	AY188355
22	PCVII-b	1767	Netherlands	AY484410
23	PCVII-b	1767	UK	AY484414
24	PCVII-b	1767	Germany	AF201311
25	PCVII-b	1767	Canada	FJ655419
26	PCVII-b	1767	Canada	FJ655418
27	PCVII-b	1767	Denmark	FJ935780
28	PCVII-b	1767	USA	GU799576

**Table 7:** Swine genes used in this study

**No**.	**Accession no**.	**No**.	**Accession no**.
1	CU405721	11	NM_001170768
2	FP016027	12	EU650400
3	CU207381	13	NM_214192
4	CU929665	14	NM_001206456
5	CU462996	15	NM_001206454
6	HM107780	16	NM_001206449
7	HM107778	17	NM_001206446
8	NM_001171541	18	NM_001206443
9	EF619476	19	NM_001206431
10	EF619475	20	FP015910

**Table 8:** Viral sequence of Circoviridae used in this study

No	Virus	Isolation	Accession number
1.	Beak and feather disease virus	South Africa	HM748939
2.		South Africa	HM748938
3.		Portugal	GU047347
4.		Portugal	EU810207
5.		Japan	AB277749
6.	Chicken anemia virus	China	FR850030
7.		China	FR850028
8.		China	FR850023
9.		Malaysia	AF390038
10.		Malaysia	AY040632
11.		China	DQ141673
12.	Columbid circovirus	America	DQ915962
13.		France	DQ915960
14.		Australia	DQ915959
15.		Belgium	DQ915958
16.		China	DQ090945
17.		Italy	DQ915950
18.	Goose circovirus	Taiwan	AF418552
19.		China	DQ192280
20.		China	GU320569
21.	Duck circovirus	China	HQ180266
22.		China	HQ180265
23.		China	GQ423746
24.		China	GQ423745
25.		China	GQ423740

### Synonymous codon usage measures

In order to eliminate the influence of amino acid composition on codon usage and directly reflect the usage characteristics of codon, the study evaluates synonymous codon usage bias through statistical estimation on relative synonymous codon usage frequency (RSCU) [37]. RSCU value refers to the ratio between the usage frequency of one codon in gene sample and expected frequency in the synonymous codon family. If the synonymous codon usage of one amino acid has no preferences, that is, codon usage frequency is close to expected frequency, the RSCU values of codons are equal to 1; if a codon RSCU value is greater than 1, the codon use frequency is higher than expected frequency, whereas it is less than expected value.

The definition on a single gene codon bias is mainly based on effective number of codons (ENC) [38]. ENC values can reflect the preference degree of synonymous codon non-equilibrium use in codon family. The range of ENC values is from 20 (each amino acid only uses one codon) to 61 (all synonymous codons are equivalently used). ENC value is closer to 20, the degree of being used non-randomly is higher, and the bias is stronger. It is generally believed that the genes are provided with significant codon bias when ENC ≤ 35. The values of RSCU and ENC were obtained by codonW program.

A comparison of actual and expected dinucleotide frequencies of the 16 dinucleotides in coding region of PCV genomes was also undertaken using SPSS 17.0.

### Correspondence analysis (CA)

Correspondence analysis is mainly used for detecting the changes of codon RSCU values in genes [39]. It is an effective multivariate statistical method of studying the internal relation between the variables and samples, and it is successfully applied to the study of codon. In correspondence analysis, all genes in samples are distributed in a 59-dimensional (59 justice codons, in addition to the stop codon, Met, and Trp) vector space, each gene is described with 59 (*f'*_1_, *f'*_2,_..., *f'_59_*) variables, the results can be applied for finding out the major factors affecting codon usage bias in genes [40,41]. This was done using the CodonW program.

### Correlation analysis

Correlation analysis of PCV was used to identify the relationship between nucleotide composition and synonymous codon usage pattern [42]. All statistical processes were carried out by with statistical software SPSS17.0 for windows.

## Competing interests

The authors declare that they have no competing interests.

## Authors' contributions

XS L and YL W conceived of the study. XS L downloaded these sequences, calculated the data, analyzed the results and drafted the manuscript; YZ F assisted with data analysis; YG Z supervised the research and helped draft the manuscript. All authors read and approved the final manuscript.
